# Charitable Institutions of Victoria

**Published:** 1905-08-05

**Authors:** 


### CHARITABLE INSTITUTIONS OF VICTORIA.

According to the report of the Inspector of the Victorian Charities, their position for the year ending June 1904, was financially sound. Their total revenue had been £280,392, against £273,606 expenditure. The overdrafts amount to £20,914, but this debt is in no way calculated to excite uneasiness. The overdraft is spread over 78 institutions, only nine of which closed the year with a debit balance of over £1,000. Moreover, the greater portion of the overdrafts being on account of building operations, the payments for which can be spread over an extended period, the indebtedness can readily be discharged by special local efforts and economies. The inspector, in commenting upon the system of overdrafts, remarks that until some power of limitation is imposed upon the bodies of the Victorian Institutions in regard to the erection of buildings of an extensive character—often unneeded—there is very little prospect of clearing off the overdrafts. The amount of municipal grant to charity during the year was £10,786, the same as the preceding year. No uniform practice prevails in the allotment of the municipal contribution, some of the local bodies giving large amounts, some smaller sums, and some no contribution at all, whilst again others levy a special rate for charitable purposes. The sum received locally from all sources is in many instances far from commensurate with the resources of the district. The only remedy is to compel the municipalities to contribute their quota to the charities on a uniform basis, and to bring the claims of the institutions more prominently before the public. There is considerable talk about the necessity for more systematic collection, but very little effort in this direction is made, though, as showing what can be done, the St. Vincent's Hospital might be quoted. Special organised efforts were made by the managers of this particular institution throughout the State to collect extra money for building purposes, and a sum of £8,000 was the reward of the year's work! That the public is not ungenerous when appealed to specially is shown by the amount brought to the coffers of the institutions by entertainments and church collections, £14,617 being produced by the former and £9,154 by the latter. There is an increase of £1,211 in the total sum made by patients' contributions, the total being £19,627, but were greater care exercised by some of the managing bodies in seeing that suitable cases for hospital treatment paid according to their means it is estimated that this source of revenue should reach at the very lowest £30,000 per annum. The inspector takes the opportunity of his report to impress upon the hospital authorities that it is not sufficient to take care that the giving of tickets of admission is limited to those in destitute circumstances and to accident cases, but they are bound also to see that those who are stated to be only capable of contributing something towards their maintenance pay strictly in proportion to their circumstances in life, and are *bonâ fide* people not in a position to pay the ordinary medical fees without having recourse to a hospital. This remark is intended to apply to out-patients, as well as to in-patients. Two Royal Commissions on the charities have brought the matter of the need for the amalgamation of the surplus institutions prominently forward. Were some of the hospitals in the Colony transformed into benevolent asylums, it is estimated that ample accommodation could be provided for both the sick and the indigent without any additional expense to the State. Such questions as these emphasise the want which has long been felt by those in the Colony interested in charity, of some central authority with power to enforce reforms in this direction. Last year in making his report, special efforts at economy were advocated by the inspector with good result. According to the statistics now to hand, in the Women's Hospital the cost per head of the inmates for the year 1903-1904 has dropped from £82 to £77; in the Melbourne Hospital, from £77 to £73; in the Eye and Ear Hospital, from £53 to £47; whilst in the Austin and Homœopathic Hospitals there has been a fall of £4 and £5 per head respectively in the yearly cost. These economies have been effected not in the food nor the medicine of the patients, but by care in the upkeep of the institutions in question.

